# Perchlorate, nitrate, and thiocyanate: Environmental relevant NIS-inhibitors pollutants and their impact on thyroid function and human health

**DOI:** 10.3389/fendo.2022.995503

**Published:** 2022-10-21

**Authors:** Caroline Serrano-Nascimento, Maria Tereza Nunes

**Affiliations:** ^1^ Instituto de Ciências Ambientais, Químicas e Farmacêuticas (ICAQF), Universidade Federal de São Paulo (UNIFESP), Sao Paulo, Brazil; ^2^ Laboratório de Endocrinologia Molecular e Translacional (LEMT), Universidade Federal de São Paulo, Sao Paulo, Brazil; ^3^ Department of Physiology and Biophysics, Institute of Biomedical Sciences, University of Sao Paulo, Sao Paulo, Brazil

**Keywords:** NIS, perchlorate, nitrate, thiocyanate, thyroid

## Abstract

Thyroid disruptors are found in food, atmosphere, soil, and water. These contaminants interfere with the thyroid function through the impairment of thyroid hormone synthesis, plasma transport, peripheral metabolism, transport into the target cells, and thyroid hormone action. It is well known that iodide uptake mediated by the sodium-iodide symporter (NIS) is the first limiting step involved in thyroid hormones production. Therefore, it has been described that several thyroid disruptors interfere with the thyroid function through the regulation of NIS expression and/or activity. Perchlorate, nitrate, and thiocyanate competitively inhibit the NIS-mediated iodide uptake. These contaminants are mainly found in food, water and in the smoke of cigarettes. Although the impact of the human exposure to these anions is highly controversial, some studies indicated their deleterious effects in the thyroid function, especially in individuals living in iodine deficient areas. Considering the critical role of thyroid function and the production of thyroid hormones for growth, metabolism, and development, this review summarizes the impact of the exposure to these NIS-inhibitors on thyroid function and their consequences for human health.

## Introduction

Several chemicals widely present in the environment affect important biological functions through the disruption of the endocrine system. One classical example of these disruptive phenomena was the discovery of intersex fish in English rivers, which was shown to be related to contaminants that interfere with estrogens action (1). Since then, the knowledge in this field has significantly progressed, and many studies have been published about the endocrine disruptors and their impact to human and other animals’ health (2). These important studies are now influencing worldwide regulation and public health policies to mitigate the potential deleterious impacts of the endocrine disruptors in the health and survival of different species.

Although there is a massive progress in the description of new molecules with endocrine disrupting properties, one great challenge in this area is to evaluate the consequences of endocrine disruptors mixtures (3) In fact, the exposure of humans and other animals to complex mixtures of endocrine disruptors complicates the determination of safe levels of exposure (4). In addition, many studies demonstrated that the continuous exposure to low doses of the endocrine disruptors affect several physiological functions (5) Thus, there are several aspects that need to be considered to define the real impact of the exposure to these contaminants on the human health.

It is well known that thyroid hormones play crucial roles in the control of metabolism, normal development, growth, and differentiation processes (6). Therefore, great attention has been given to the thyroid disruptors. Indeed, it has been reported that these disruptors affect several steps of thyroid physiology, like thyroid hormones synthesis, transport, action, and peripheral metabolism (7).

This mini review is centered on anions classified as environmental contaminants that affect thyroid hormone synthesis, particularly those that interfere with the activity of sodium iodide symporter (NIS), which mediates the iodide uptake, the first and limiting step for the thyroid hormone synthesis (8)NIS is expressed at the basolateral membrane of the thyroid follicular cells, as well as in non-thyroidal tissues, like mammary, salivary, and lacrimal glands, stomach, and small intestine (9)In summary, the data about the impact of perchlorate, nitrate and thiocyanate exposure on thyroid function and human health will be addressed herein.

## Perchlorate

Perchlorate (ClO4^-^) is a strong oxidizing anion used in rockets fuels, explosives, fireworks, and missile fuels. Perchlorate is also naturally formed in the atmosphere and is accumulated in arid climate regions (10). In fact, human manufacturing of perchlorate-containing products and the naturally formed perchlorate result in a large occurrence of this compound in the environment, as demonstrated by its presence in irrigation water and soil. Perchlorate is also present in some fertilizers, increasing its accumulation in fruits and vegetables (11) Therefore, human exposure to perchlorate mainly occurs from contaminated food and drinking water.

Perchlorate is a potent inhibitor of iodide uptake mediated by NIS in the thyrocytes, impairing the first step of thyroid hormone synthesis (12). This inhibitory effect has been recognized since the 50’s, when perchlorate was commonly used as a therapeutic drug for treating hyperthyroidism (13). Moreover, since this anion displaces iodide from the thyroid, it was used in the “perchlorate challenge” test, for the detection of thyroid iodine organification defects (14).

Several studies contributed to characterize the disruptive effects of perchlorate on thyroid function and to reinforce the importance of the regulation of perchlorate levels in the environment. Perchlorate and iodide are anions with similar charge and size. However, it is worth noting that NIS has a higher selectivity for perchlorate than for iodide. In agreement, *in vitro* studies have shown that iodide transport is essentially abolished in thyrocytes exposed to 10 µM of perchlorate, without alterations in the expression of NIS (12) for many years, it was suggested that perchlorate was a potent inhibitor of NIS-mediated iodide uptake without being transported into the thyrocytes (15). However, elegant studies demonstrated that this anion is actively transported by NIS in an electroneutral stoichiometry (16, 17). Besides the potent inhibition of NIS activity, it has been described that perchlorate also suppresses the thyroglobulin and thyroperoxidase gene expression, which was associated with the impairment of the thyroid hormone synthesis induced by this contaminant (18).

The deleterious effect of perchlorate exposure were previously demonstrated in species that depend on thyroid hormone action to drive their metamorphosis processes, as amphibians and fishes. In accordance, several abnormalities in the development, reproduction, and survival were described in perchlorate-exposed animals (19, 20). In contrast, the exposure to perchlorate has not altered the metamorphosis or the thyroid histopathology of common frogs (21).

In humans, the effects of perchlorate on thyroid function are still controversial. Several studies reported that perchlorate exposure was not associated with alterations in TSH or T4 serum levels in humans (22–24). However, other studies have shown significant alterations in the function of the pituitary-thyroid axis in humans co-exposed to perchlorate and other NIS-inhibitory anions, such as nitrate and/or thiocyanate, especially, but not exclusively, in iodine deficient areas (25, 26). A recent study has also indicated that humans co-exposed to perchlorate, nitrate and thiocyanate presented an increased central thyroid hormone sensitivity, which seem to be more precise than the single parameters, as TSH or T4 serum levels, to evaluate the homeostasis of the pituitary-thyroid axis (27).

It has been suggested that the disruptive actions of perchlorate on thyroid function are more critical during specific windows of susceptibility, as the pregnancy. Even so, the consequences of maternal exposure to perchlorate in the thyroid function are still controversial (28–31). In fact, the different conclusions about the deleterious effects of perchlorate exposure during this critical developmental period are related to the different ranges of exposure in different human populations as well as to the period of the evaluation in each study. Therefore, more studies are needed to further clarify this issue. Conversely, a study focused on pregnant women with borderline thyroid function living in iodine deficient areas demonstrated that perchlorate exposure impaired the offspring cognitive development. This impairment was not reversed by maternal levothyroxine therapy, suggesting that the fetal thyroid function is more susceptible to the perchlorate-induced disruption (32). In agreement, the offspring rats of pregnant rats exposed to perchlorate presented several alterations in the synaptic function during adulthood (33).

An elegant study from the group of Dr. Nancy Carrasco demonstrated that perchlorate is transported to maternal milk through the activity of NIS that is expressed in the mammary glands (17). Consequently, besides the reduction of iodide transferred to the milk, the newborns could be exposed to high levels of this potent NIS inhibitor, which could potentially impair the central nervous system development, since it is highly dependent on thyroid hormone action (34, 35).

Furthermore, studies have suggested a positive association between perchlorate exposure and the risk to develop papillary thyroid cancer (36, 37). As the incidence of thyroid cancer is increasing worldwide, the contribution of the thyroid disruptors, such as the perchlorate, which has a potent disruptive action on NIS activity and on thyroid function should be addressed.

Finally, in rodents, the chronic exposure to ammonium perchlorate through drinking water altered the serum levels of thyroid hormones and TSH serum levels and the morphology of the thyroid gland (38) Our studies reinforced these data and described some of perchlorate-induced molecular mechanisms involved in the disruption of the hypothalamus-pituitary-thyroid axis. Indeed, the animals exposed to perchlorate presented primary hypothyroidism, as shown by the decreased serum T4 and T3 levels, and increased serum TSH concentration. Additionally, the exposure to perchlorate induced alterations in the expression of genes/proteins involved in the thyroid hormone synthesis and increased several markers of inflammation in the exposed animals (39).

Interestingly, it has been shown that TSH increases the NIS-mediated perchlorate transport into the thyroid cells (16). Furthermore, it has been previously described that perchlorate *per se* induces a unique pattern of gene expression alterations in the thyroid gland, that is completely different from the one induced by iodine deficiency (40) Even though, the molecular mechanisms involved in the regulation of thyroid gene expression need to be further clarified.

Thus, although some studies indicate the potential deleterious effects of perchlorate exposure on thyroid function, there are many controversial results, especially in epidemiologic studies. This reinforces the necessity of more studies to clarify the period as well as the doses of exposure to perchlorate that are potentially more harmful to the health of humans and other animals.

## Nitrate

Nitrate (NO3-) is a naturally occurring anion in the environment since it is part of the nitrogen cycle. The plants obtain nitrogen, an essential component for the synthesis of plant proteins, through the absorption of nitrate from the soil and the groundwater (41). Therefore, humans are mainly exposed to nitrate through the consumption of green leafy vegetables, roots, oilseeds, grains, tubers, and nuts. Moreover, nitrate is commonly found in agricultural fertilizers and in preservative additives for cured meats. Accordingly, the presence of nitrate in the environment is greater than the one observed for thiocyanate or perchlorate.

The nitrate levels in the drinking water sources and food have significantly increased in recent decades due to the exacerbated use of nitrogen fertilizers. Alarmingly, the effective nitrate removal from water sources depends on complex and highly costly processes, which are rarely performed (42). In agreement, studies suggest that nitrate concentration in food and water sources will highly increase in the future, due to the increased use of nitrogen fertilizers and the intensification of agricultural activities to support human population growth (43). It is worth noting that the maximum contaminant level of nitrate in the drinking water was defined by the U.S. Environmental Protection Agency (EPA) and the World Health Organization (WHO) as 10 mg/L for nitrate-nitrogen, which is equivalent to 45 mg/L as nitrate (44). Nevertheless, it has been demonstrated that some regions of the world present higher concentrations of nitrate in the water, which greatly exceeds the levels considered safe for human exposure (45).

Interestingly, some types of cancer, as gastric and colorectal cancers, were previously associated with the exposure to nitrate at levels that are considered safe by the regulatory agencies (46, 47). The deleterious effects of nitrate have been related to its conversion into other nitrogen-containing compounds in the body (48). Thus, it is well known that nitrate is converted to nitrite, which can subsequently react with amines and amides in the gastrointestinal tract to form N-nitroso compounds (NOCs), a class of known carcinogenic and cytotoxic substances (49).

Additionally, high levels of nitrate consumption were associated with an increased risk for reproductive problems. Both nitrate and nitrites are precursors of nitric oxide (NO), a lipophilic molecule with several physiological roles. However, excessive production of NO was associated with several pathophysiological events, as reproductive system dysfunctions and impaired production of sexual steroids (50, 51). Other studies indicated that the exposure to high levels of nitrate during pregnancy is a risk factor for spontaneous abortion, fetal death, prematurity, intrauterine growth restriction, low birth weight, congenital malformations, and neonatal death (52). Therefore, besides its carcinogenic potential, the deleterious effects of nitrate on the endocrine system have received increasing attention in the recent years (53). In addition to the nitrate-induced damage to other endocrine glands, some studies suggest that nitrate exposure impairs the thyroid function in humans and other animals.

Nitrate competitively inhibits NIS-mediated iodide uptake with a much lower potency than the one induced by perchlorate (12). Nevertheless, the concentration of nitrate detected in human and environmental samples were much higher than those described for perchlorate. This fact could potentially contribute to the harmful effects induced by nitrate in the body.

Although the *in vitro* assays clearly demonstrated the inhibitory effects of nitrate on NIS function, suggesting an impairment of the thyroid function, the effects of nitrate exposure on pituitary-thyroid axis in humans are still controversial. Indeed, nitrate exposure was associated with increased risk of developing thyroid disorders, especially in susceptible individuals as pregnant women, newborns, and children, as well as in women with urinary iodine levels ≥ 100 µg/L (25, 54–56). Furthermore, chronic exposure to high levels of nitrate through public water supplies was associated with increased risk of developing thyroid cancer (57, 58). However, other studies have not detected any alteration in TSH and/or T4 serum levels in nitrate-exposed humans (31, 59).

Although the inhibition of thyroid function by nitrate is reported in the literature, the molecular mechanisms involved in this phenomenon are not completely elucidated. Indeed, the chronic exposure of male rats to high levels of nitrate increased thyroid weight, induced morphological alterations in the thyroid follicles and altered thyroid hormone production (60, 61). In accordance, a goitrogenic effect was also observed in female rats chronically exposed to nitrate through drinking water. However, no alterations in thyroid hormone or TSH serum levels were observed, which were associated with increased expression of genes involved in the synthesis of thyroid hormones (62).

As discussed before, nitrate exposure increases the production of NO in different tissues, which promotes post-translational modifications, such as nitrosylation of cysteine ​​residues and nitration of tyrosine residues that change the stability, location, and activity of several proteins (63, 64). In this sense, in thyrocytes, it has been shown that the excessive production of NO decreases the expression of transcriptional factors, such as *Foxe1*, inhibits NIS-mediated iodide uptake, and interferes with the signaling pathway triggered by TSH, which potentially inhibits the expression of different genes involved in the biosynthesis of thyroid hormones (65–68). Therefore, although some aspects related to the nitrate-induced impairment of the thyroid function have been described, future studies are needed to unravel the molecular mechanisms involved in the direct effects of this anion on the thyroid, as well as the potential deleterious effects of nitrate on the development of the thyroid gland.

## Thiocyanate

Thiocyanate (SCN-) is vastly found in food that contain thioglycosides – such as cassava, bamboo shoots, sweet potatoes, brussels sprouts, cauliflower, corn broccoli, apricots, and almonds. The inhalation of cigarette smoke in another importance source of human thiocyanate contamination since it contains cyanide, which is converted into thiocyanate in the body. Thiocyanate is highly soluble in water and previous data demonstrated a relevant contamination of the groundwater with this anion (69). It is worth noting that the half-life of thiocyanate is approximately 6 days, much longer than the few hours half-lives presented by perchlorate and nitrate.

Thiocyanate has a goitrogenic action since it inhibits NIS-mediated iodide uptake (12). Electrophysiological studies indicated that thiocyanate is transported by NIS into the thyrocytes with a similar stoichiometry of I^-^ (15, 70). Similar to nitrate, the potency of the thiocyanate-mediated inhibition of NIS activity is lower than the one exerted by perchlorate (12). Nevertheless, epidemiologic studies reported higher levels of thiocyanate in comparison to perchlorate in the serum of the US population (25) In addition, studies demonstrated that besides the inhibitory effect on NIS activity, thiocyanate impairs the iodine organification catalyzed by the thyroperoxide (71, 72).

Thiocyanate is also transported to the milk of breastfeeding mothers who smoke cigarettes. Alarmingly, increased levels of thiocyanate in the maternal milk were correlated with decreased iodide content in the milk. As expected, this condition was associated with higher risk of developing thyroid dysfunctions in the newborns, due to the direct exposure of these individuals to a potent NIS-inhibitor and to decreased levels of the main substrate for the thyroid hormones synthesis (73). Furthermore, epidemiological studies indicated that thiocyanate exposure was associated with the inhibition of thyroid hormone production and the development of thyroid autoimmunity (74, 75). However, although previous studies indicated that the co-exposure to perchlorate and thiocyanate is potentially deleterious to the thyroid function in adults and in susceptible individuals, as pregnant women, fetus, and newborns, the effects of thiocyanate *per se* in the thyroid are still controversial in humans (27, 30, 31, 76)

Finally, the molecular mechanisms involved in the thiocyanate regulation of thyroid function are not completely understood. Studies using primary thyroid cells cultures exposed to plant extracts rich in thiocyanate demonstrated an increased production of reactive oxygen species, induced cell injury and DNA damage, decreased gene expression and activity of proteins involved in the synthesis of thyroid hormones (77, 78). However, the direct effects of thiocyanate *per se* in the thyrocytes have never been reported.

## Current gaps and future perspectives

**Figure 1:**
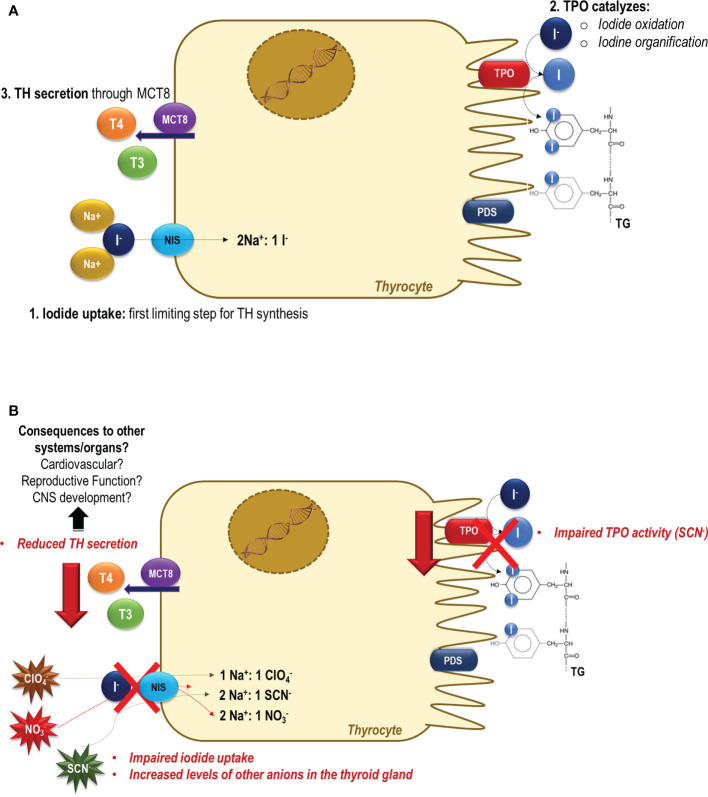
Thyroid dysfunction induced by perchlorate, nitrate and/or thiocyanate exposure. **(A)** In a normal condition, the sodium-iodide symporter (NIS) mediates the iodide uptake, the first limiting step for thyroid hormone synthesis. Then, iodide is transported across the apical membrane, and it is oxidized and organified into tyrosyl residues of thyroglobulin (TG) through the activity of thyroid peroxidase (TPO). Under the TSH stimulus, thyroid hormones (TH) are secreted and exert their effects in several tissues/organs, controlling the metabolism, growth, and development (79). **(B)** In the presence of perchlorate, nitrate and/or thiocyanate, NIS-mediated iodide uptake is impaired, and these molecules are actively transported into the thyroid cells (12). The consequences of increased levels of these anions in the intracellular thyrocytes medium are still unclear. It has been reported that besides the inhibition of NIS activity, the SCN- also impairs the organification of iodine catalyzed by TPO activity. The impairment of the activity of these two key proteins involved in the thyroid hormone synthesis could contribute to the reduced production and secretion of thyroid hormones to the blood circulation. The negative consequences of the reduction of the thyroid hormone serum levels are widely described in the literature, but are especially alarming during critical periods of the development, as during the pregnancy and lactation periods.

Several studies have been carried out in recent years and their results have clarified many aspects of the deleterious effects promoted by the exposure of animals to perchlorate, nitrate, and thiocyanate (**Figure 1**). Nevertheless, there are several aspects and molecular mechanisms that need to be clarified. Indeed, especially the epidemiological data are still controversial, and the impact of the exposure to these NIS-inhibitors on human health are not conclusive. The controversial results may be related to the different methodologies that were used to determine these contaminants in each study, as well as the period of the exposure that was evaluated. Moreover, these anions have a short biological half-life, which could impair these associative analyzes. Additionally, there are no data about the long-term and programming-induced effects of these contaminants, especially during the windows of susceptibility, such as pregnancy and lactation. In general, the harmful effects of these contaminants were observed in iodine-deficient populations and were related to the induction of iodine deficiency. This concern is irrefutable, however there are scarce data about the direct effects of these contaminants on the thyroid gland. It is important to highlight that NIS is expressed in several other tissues as mammary glands, placenta, intestine, kidney, gonads. Therefore, future studies are needed to clarify the effects of these anions in other systems/organs besides the hypothalamus-pituitary-thyroid axis. Finally, it is important to reinforce that the disruption of thyroid function goes beyond the impairment of the synthesis of thyroid hormones, since these hormones affect virtually all organs/systems of the body.

## Author contributions

All authors listed have made a substantial, direct, and intellectual contribution to the work and approved it for publication.

## Funding

CS-N is supported by a grant from Fundação de Amparo à Pesquisa do Estado de São Paulo (FAPESP: 16/18517-8). MN was supported by grants from FAPESP (13/05629-4) and CNPq (310473/2021-7).

## Conflict of interest

The authors declare that the research was conducted in the absence of any commercial or financial relationships that could be construed as a potential conflict of interest.

## Publisher’s note

All claims expressed in this article are solely those of the authors and do not necessarily represent those of their affiliated organizations, or those of the publisher, the editors and the reviewers. Any product that may be evaluated in this article, or claim that may be made by its manufacturer, is not guaranteed or endorsed by the publisher.
